# Experimental investigation on a solar parabolic collector using water-based multi-walled carbon-nanotube with low volume concentrations

**DOI:** 10.1038/s41598-023-34529-6

**Published:** 2023-05-06

**Authors:** Vinayak Talugeri, Nagaraj Basavaraj Pattana, Veeranna Basawannappa Nasi, Kiran Shahapurkar, Manzoore Elahi Mohammad Soudagar, Tansir Ahamad, Md. Abul Kalam, Kiran Madrahalli Chidanandamurthy, Nabisab Mujawar Mubarak, Rama Rao Karri

**Affiliations:** 1 Department of Mechanical Engineering, M. S. Ramaiah Institute of Technology (Affiliated to VTU), Bangalore, 560054 India; 2 Department of Mechanical Engineering, School of Mechanical, Chemical and Materials Engineering, Adama Science and Technology University, 1888, Adama, Ethiopia; 3 Department of VLSI Microelectronics, Saveetha School of Engineering, Saveetha Institute of Medical and Technical Sciences, Chennai, Tamilnadu 602105 India; 4 Department of Chemistry, College of Science, King Saud University, Riyadh, Saudi Arabia; 5 School of Civil and Environmental Engineering, FEIT, University of Technology Sydney, Ultimo, NSW 2007 Australia; 6 Department of Mechanical Engineering, Nitte Meenakshi Institute of Technology, Yelahanka, Bangalore, 560064 India; 7 Petroleum and Chemical Engineering, Faculty of Engineering, Universiti Teknologi Brunei, Bandar Seri Begawan, BE1410 Brunei Darussalam

**Keywords:** Energy science and technology, Engineering, Materials science, Nanoscience and technology

## Abstract

A limited experimental work was on multi-walled carbon nanotube (MWCNT)—water nanofluid with surfactant in the solar parabolic collector at low volume concentrations. At high-volume concentrated nanofluid, the pressure drop was more due to an increase in the viscosity of the working fluid and an increase in the nanoparticle cost; hence it is not economical. This report attempted to use Sodium Dodecyl Benzene Sulfonate (SDBS) surfactant in the low-volume concentrated MWCNT-water nanofluid to establish effective heat transfer in solar parabolic collector applications. The stable MWCNT-water nanofluid was prepared at 0.0158, 0.0238, and 0.0317 volume concentrations. The experiments were conducted from 10:00 to 16:00 at 6, 6.5 and 7 L/min flow rates concerning ASHRAE Standards. At the 7 L/min flow rate of the working fluid, having a minimum temperature difference between the working fluid and absorber tube leads to better heat transfer. The increased volume concentration of MWCNT in the water enhances the surface area interaction between water and MWCNT nanoparticles. This results in maximum solar parabolic collector efficiency at 0.0317 vol% with a 7 L/min flow rate and 10–11% higher than the distilled water.

## Introduction

The increase in energy demand and issues like global warming and hazardous emissions of fossil fuels resulted in shifting toward renewable energy sources. Solar energy was one of the promising options to meet the present energy needs. Solar energy can be derived from solar collectors and photovoltaic cells. Photovoltaic cells directly convert solar energy into electric energy and solar collectors are used for higher-temperature applications The parabolic collector is a linear concentrator-type solar collector which operates at 150–400 °C^1^. The parabolic collector consists of a mirror or collector, which reflects the solar radiation and has the shape of a parabola, and an absorber tube or receiver tube, which receives the radiation from the mirror and is located at a focal position of the mirror. The absorber tube transfers heat to the working media. This heated fluid is utilized for industrial and power generation applications. Modifying the receiver tube and working media enhances the heat transfer in a parabolic collector. Modifying the receiver tube means changing the material for the receiver tube, applying thermal coating on the receiver tube, modifying the receiver tube's design, changing the receiver tube's internal face, and adding an effective glass cover to the external face of a receiver tube. The higher thermal conductivity materials were chosen for the receiver tube. The advancement of the working fluid can be accomplished by the introduction of nanoparticles to the base fluid, and such a fluid is known as nanofluids. In nanofluids, the nanoparticle's role is to enhance heat transfer by increasing the thermal conductivity of a working fluid. Hence, higher thermal conductivity nanoparticles are used in nanofluids. Many researchers have worked on the effect of the volume concentration of nanofluid, volume flow rate, and absorber tube material on the performance of solar parabolic collectors. The influence of weather conditions and the intensity of solar radiation is also accounted for. A detailed literature review was carried out on the parameters listed above, which are discussed below. The experiments were carried out for different coatings and materials of the receiver tube using volume fractions of 0.2 and 0.3 vol% CNT-oil as a Functioning fluid. The experiments were conducted with a parabolic collector to check the optical and thermal performance of the absorber tube. They found that the black chrome-plated vacuumed copper tube produced good results^2^. The experimental work was carried out by coating 20–40 nm CNT nanoparticles on the copper absorber tube.

The result shows that modification of coated CNT nanoparticles on an absorber tube at 0.05 vol% of Al_2_O_3_ nanofluid with 2 L/min obtained 8.6% collector efficiency enhancement compared to distilled water^3^. The numerical analysis was carried out on the U-shaped receiver tube of the parabolic collector with a hybrid nanofluid and volume fraction varying from 1 to 4%. The simulation was based on the Eulerian-Eulerian method for simulating surface multi-phase nanofluid flow, face-to-face interaction for the simulation of radiation and a typical k-turbulence model used for turbulent calculations. The results showed that the U-shaped receiver tube gave better results than a standard pipe with the same hydraulic diameter^4^. A numerical investigation has been carried out on a converging–diverging receiver tube with an Al_2_O_3_-thermal oil nanofluid. The converging–diverging sine wave geometry receiver tube increased the surface area of heat transfer compared to a cylindrical absorber tube. This results in more turbulence in the flow. This turbulence enhances heat transfer and collector efficiency^5^. The experiments have been carried out on the influence of the glass tube covering the absorber tube on optical performance. The receiver tube consists of a glass tube on the outside, which enhances the transmissivity with long-wave radiation and increases the collector's optical performance compared to the bare receiver tube. The investigation was done by varying the volume concentration for 0.1, 0.2 and 0.3 vol% MWCNT in ethylene glycol^6^. An experimental investigation has been carried out on the absorber tube by inserting the twisted nail tape. The Al_2_O_3_–H_2_O nanofluid was used as working fluid at 0.1 and 0.3% volume concentrations in a solar parabolic collector. The laminar flow conditions were considered during the study and analyzed the heat transfer and friction factors. The result concluded that the presence of twisted nail tape in the absorber tube with nanofluids performs a significant heat transfer and, at the same time, increases the friction factor^7^. The effect of modification in the absorber tube is explained in the research study.

An experimental investigation was conducted with Al_2_O_3_–water nanofluid in the solar parabolic collector by varying the volume fraction from 0.05 to 0.5% and mass flow rate from 0.0083 to 0.05 kg/s. The highest solar collector efficiency was achieved at 0.05 kg/s with 0.5 vol% of Al_2_O_3_. The affixing of nanoparticles in the base fluid improves the collector efficiency from 3.4 to 8.54% concerning water^8^. An experimental and numerical investigation has been conducted on MWCNT-water nanofluid in a solar parabolic collector at different locations. The result concluded that low-volume concentrations form better thermo-hydraulic performance for flow rates less than 0.2 L/s^9^. The experimental study was conducted on graphene oxide and alumina nanoparticles at 0.2 wt% in a water-based nanofluid. The solar parabolic system's flow rate varied from 1 to 5 L/min. The best collector efficiency of 63.2% was found at 1 L/min using graphene oxide–water nanofluid compared to pure water. This result was due to graphene oxide nanoparticles being more elongated than alumina and forming a thin layer on the inner surface of the absorber tube to avoid bubble formation for better heat transfer^10^. A numerical analysis was carried out on CuO and Al_2_O_3_ nanoparticles at a 3% quantity fraction in water for a solar parabolic collector. The finite volume approach was adopted for evaluation using the k–ε RNG turbulent model for distinct warmth inputs. The numerical examination confirms that the warmth switch increased by 28% for Al_2_O_3_-water and 35% for CuO-water nanofluids at 3 vol%^11^. The above research work gives information about the effect of different nanoparticles and their concentrations on the performance of solar collectors.

An experimental study was conducted with MWCNT-water nanofluid on a solar parabolic collector. During the experiment, the volume fraction of MWCNT turned into 0.01 and 0.02%, the water flow rate was varied as 100 and 160 L/h, and Triton X-a hundred surfactant turned into used to enhance the stableness of MWCNT. The most collector performance turned into accomplished at 0.02 vol% at 160 L/h^12^. The experimental and CFD analyses were conducted on SiO_2_–water and CuO–water nanofluids in solar parabolic collectors. The volume flow rate varied as 40 L/h and 80 L/h at 0.01 vol% of SiO_2_ and CuO nanoparticles. The stability of nanoparticles was enhanced by hexa-decyl-trimethyl-ammo-niumbromide surfactant. The experimental and CFD results concluded that CuO–water nanofluid performed better in both flow rates^13^. The effect of surfactant on the stability of the nanoparticles addresses in the above research papers.

The Mathematical model has been developed with a hybrid nanofluid in a solar parabolic collector. In hybrid nanofluids, more than one type of nanoparticle is used in the base fluid. This numerical study used 1 to 4 vol% of Ag–ZnO, Ag–TiO_2_ and Ag–MgO hybrid nanoparticle combinations in Syltherm 800 base fluid between Reynolds numbers 10,000 and 80,000. The study revealed that hybrid nanofluids are more effective than base fluid, and among all hybrid combinations, 4 vol% Ag–MgO–Syltherm 800 nanofluid has the highest thermal efficiency^14^. An experimental study has been conducted on CuO-MWCNT-water hybrid nanofluid, and results were compared with individual nanofluid. The concentrations of CuO are used at 0.15 wt% and MWCNT at 0.005 wt% in direct solar energy harvesting systems. The performance of nanofluids was analyzed by photo-thermal absorption. The result concluded that hybrid nanofluid performs better than individual nanofluids in transferring heat^15^. A numerical study was carried out on parabolic collector with the Al_2_O_3_ (3 vol%)–Syltherm 800, Al_2_O_3_ (3 vol%)–Syltherm 800, and Al_2_O_3_ (1.5 vol%)–TiO_2_ (1.5 vol%)–Syltherm 800 hybrid nanofluid. The flow rate was 150 L/min, and the inlet temperature was 300–650 K. The numerical results confirmed that hybrid nanofluids perform better than individual nanofluids^16^. The above research describes the impact of hybrid nanofluids on the efficiency of the solar parabolic collector.

To enhance the storage capacity by employing the suitable phase change material in solar collectors. The investigation analyzed single-unit solar collectors that use phase change materials (PCMs) for solar water and air heaters, examining different designs of PCM-based systems. An effective system for storage was suggested based on the applications^17^. A study tested shape-stabilized phase change material in a tankless solar water heater, improving thermal efficiency from 66 to 82%. Flow rate changes had minimal impact. Cost analysis showed a 6-year payback period and 5.4-ton annual CO_2_ emissions reduction^18^. A study investigated using a back pipe in vacuum tube solar collectors to remove stagnant regions and improve thermal performance. Results showed a 42% decrease in heat losses and a 10% improvement in heat transfer rate. Regression techniques were used to model system performance, with a reasonable agreement with experimental data^19^. These papers provided the effect of the phase change storage tank used in solar collectors.

The above discussion shows that the MWCNT nanoparticle possesses higher thermal conductivity when compared with other nanoparticles. Fewer attempts have been made to use MWCNT and surfactant to improve the performance of solar parabolic collectors. In the present work, an attempt is made to improve the performance of the parabolic collector by using MWCNT-water nanofluid, and SDBS surfactant was used to stabilize MWCNT in the distilled water. The volume fraction of MWCNT was varied as 0.0158, 0.0238 and 0.0317 vol%, and the volume flow rate was varied as 6, 6.5 and 7 L/min.

## Materials

This section reports the materials and methods used during the experimentation.

The base fluid: Distilled water and Nanoparticle: MWCNT supplied by Ad-nano technologies private limited Shimoga, Karnataka, India. The procured MWCNT was synthesized by using the chemical vapor deposition technique. The MWCNT sample underwent multiple washes with deionized water, followed by filtration and subsequent drying at 80 °C for 2 h to purify and remove the moisture^20^. Then the MWCNT sample was prepared for TEM analysis as per the standards. The TEM is a powerful tool for the characterization and analysis of nanoparticles in nanofluids, providing valuable information about their size, morphology, dispersion, crystal structure, chemical composition, interfacial interactions, and behavior under different conditions. This information is crucial for understanding nanofluids' properties and behavior and optimizing their performance in various applications.

Surfactant: To stabilize MWCNT in the distilled water, SDBS provided by Lob Chemie Pvt. Ltd. Mumbai, Maharashtra, India, was used.

### Preparation of MWCNT-water nanofluid

#### Instruments used for preparing nanofluid and experimental conduction of solar parabolic collector

**Figure 1:**
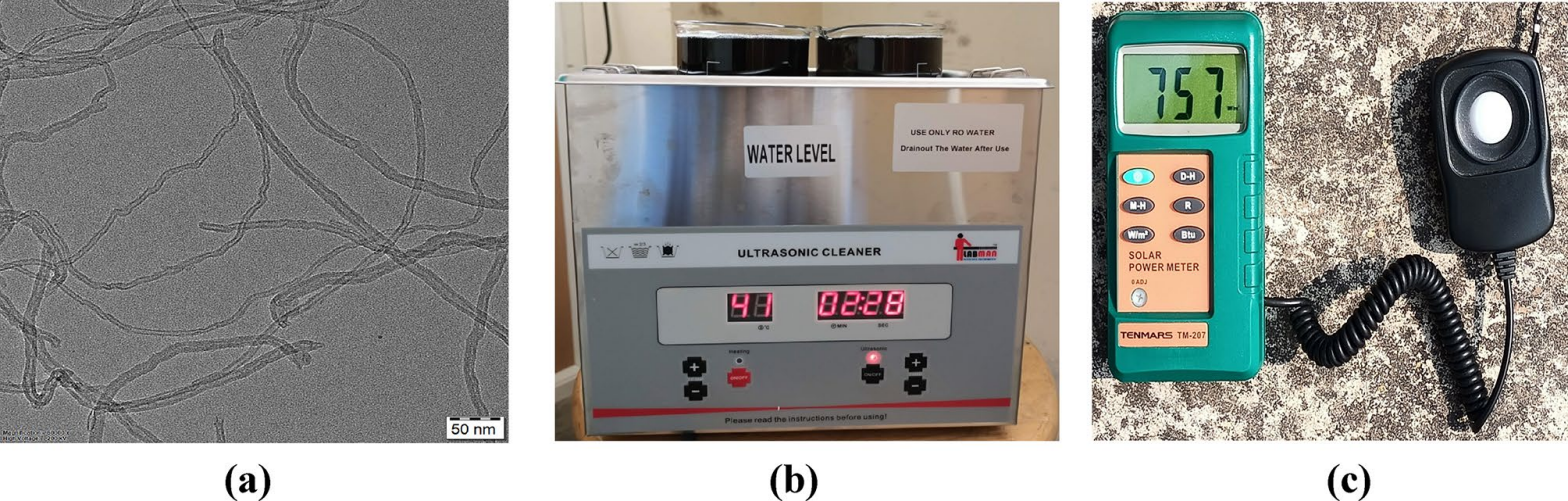
The characterization, instrument used for the preparation of nanofluid and measurement of solar radiation (**a**) MWCNT TEM image (**b**) Ultrasonic bath for nanofluid (**c**) solar power meter.

The procured MWCNT was characterized by a high-resolution transmission electron microscope to verify the physical characteristics and is shown in Fig. 1a. The diffusion of MWCNT in the distilled water was operated by using an ultrasonic bath (Labman Scientific Instruments Pvt. Ltd.) at a frequency of 40 ± 3 kHz and shown in Fig. 1b. To measure temperatures, thermocouples used and an operating range of 0–199 °C. The volume flow rate of the working fluid was measured by a rotary-type flow meter with an operating range of 1–30 L/min. To measure wind speed, the anemometer was used with a range of 0–45 m/s. The day's solar radiations were measured using a solar power meter and shown in Fig. 1c. The operating range of 0–1999 W/m^2^.

#### Preparation method of nanofluid

A two-step method was considered for the preparation of CNT nanofluid. First, a surfactant solution was prepared using Sodium-Dodecyl-Benzene-Sulfonate (SBBS), then MWCNT nanoparticles were mixed with the solution, and a 1:1 ratio of surfactant/MWCNT was maintained^21–23^. The prepared solution was applied for magnetic steer for up to 10 min at 500 rpm and then treated with ultra-sonicated for 40 min to disperse the MWCNT in the solution. The MWCNT volume fractions 0.0158, 0.0238, and 0.0317 vol% were used during the formation of the nanofluid Table 1. Thermophysical properties of MWCNT and water were considered during the calculation. The stability of the MWCNT was further discussed in Section "Effect of different concentrations of MWCNT nanoparticles in the water".

**Table 1:** Thermo-physical properties of water and MWCNT^24^.

Properties	MWCNT	Water
Density at 15 °C (kg/m^3^)	2100	998.95
Thermal conductivity at 35 °C (W/m K)	2000	0.626
Specific heat capacity at 45 °C (J/kg K)	733	4174

### Thermophysical properties of nanofluids

As the nanoparticles were added to the base fluid water, the properties of that fluid changed. The addition of the nanoparticles was measured in terms of volume concentration, which is determined by Eq. (1)^20^.

1 $$\upphi =\left[\frac{\frac{{\text{m}}_{\text{np}}}{{\uprho }_{\text{np}}}}{\frac{{\text{m}}_{\text{np}}}{{\uprho }_{\text{np}}}+\frac{{\text{m}}_{\text{bf}}}{{\uprho }_{\text{bf}}}}\right]$$

The density of the nanofluid has been formulated by Pak and Xuan model Eq. (2)^24^**.**

2 $${\uprho }_{\text{nf}}=\upphi {\uprho }_{\text{np}}+\left(1-\upphi \right){\uprho }_{\text{bf}}$$

The specific heat of the nanofluid has been calculated using Eq. (3)^24^**.**

3 $${\text{C}}_{{\text{p}}_{\text{nf}}}=\upphi {\text{C}}_{{\text{p}}_{\text{np}}}+(1-\upphi ){\text{C}}_{{\text{p}}_{\text{bf}}}$$

The thermal conductivity of the nanofluid was estimated through Maxwell model Eq. (4)^21^**.**

4 $${\text{k}}_{\text{nf}}=\frac{{\text{k}}_{\text{bf}}\left[{2\text{k}}_{\text{bf}}+{\text{k}}_{\text{np}}+2\upphi \left({\text{k}}_{\text{np}}-{\text{k}}_{\text{bf}}\right)\right]}{{2\text{k}}_{\text{bf}}+{\text{k}}_{\text{np}}-\upphi \left({\text{k}}_{\text{np}}-{\text{k}}_{\text{bf}}\right)}$$

Dynamic Viscosity of the nanofluid was determined by Bachelor model Eq. (5)^25^.

5 $${\upmu }_{\text{nf}}={\upmu }_{\text{bf}}\left({1+2.5\upphi +6.5\upphi }^{2}\right)$$

## Experimental procedure and equations for the solar parabolic collector

### Experimental setup and working of the solar parabolic collector

**Figure 2:**
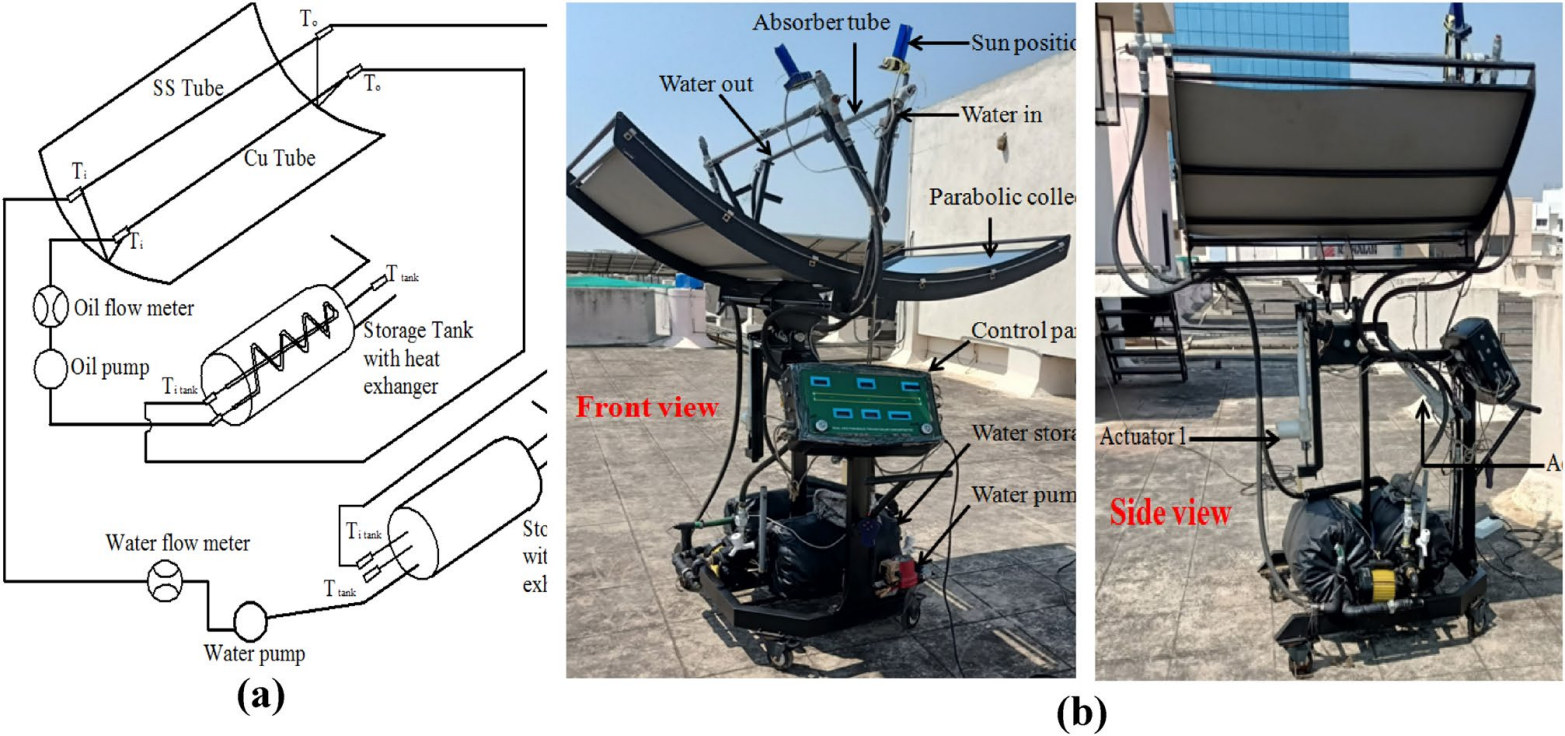
The experimental setup of the solar parabolic collector (**a**) Schematic setup of the solar parabolic collector (**b**) Test rig of the solar parabolic collector front view and side view.

The Schematic arrangement of the solar parabolic collector is shown in Fig. 2a. The performance test on the solar parabolic collector supplied by Eco-sense, Delhi, India, is conducted in Fig. 2b. Table 2. provides the details of the specifications of the equipment. The experiment was piloted in Bengaluru, India (13° 1′ 50″ N–77° 33′ 54″ E). The solar collector was positioned north–south, and the setup has an auto-tracking mechanism to adjust the position of the parabolic collector with the sun's position every 20 min. The parabolic collector has a concentration factor of 20, representing the amount of solar radiation concentrated on the absorber tube, with a reflectivity of 85% and an absorptivity of 95%. The higher concentration factor, reflectivity and absorptivity increase the heat transfer of the working fluid by attaining a higher temperature. The experiments were conducted as per ASHRAE standards. Different radiation incidents, ambient temperature, and fluid consumption temperature are considered while comparing the solar collector's overall thermal performance. A steady-nation experiment is important to decide the charge of solar radiation incident at the collector and the charge of electricity switch to the operating fluid because it flows through the collector. The experimental data were taken from 10:00 to 16:00 at every 1-h interval for a single flow rate, and the flow rate varied as 6, 6.5, and 7 L/min.

**Table 2:** Specification of solar parabolic collector setup.

Length of Parabolic trough collector	1.2192 m
Length of Parabola aperture	1.6764 m
Parabolic collector material	Acrylic mirror
Focal distance	0.6065 m
Rim Angle	67.24°
Receiver tube inner tube diameter	0.023 m
Receiver tube outer tube diameter	0.025 m
Receiver tube material	Stainless steel

### Equation of thermal efficiency in solar parabolic collector

The inlet temperature of the working fluid was different at different periods; hence the rate of useful energy gain was calculated by Eq. (6)^10^**.**

6 $${\dot{\text{q}}}_{\text{u}}=\dot{\text{m }}{\text{C}}_{\text{p}}\left({\text{T}}_{\text{o}}-{\text{T}}_{\text{i}}\right)$$

Equation (7) to determine the rate of useful energy gain was a change in gained energy and energy loss by the receiver tube^10^**.**

7 $${{\dot{\text{q}}}}_{{\text{u}}} = {\text{F}}_{{\text{R}}} ({\text{W}} - {\text{D}}_{0} )L\left\{ {{\text{S}} - {\text{F}}_{{\text{R}}} {\text{U}}_{{\text{l}}} \frac{{\left( {{\text{T}}_{{\text{i}}} - {\text{T}}_{{\text{a}}} } \right)}}{{\text{S}}}} \right\}$$

The instantaneous efficiency of the solar parabolic collector has been computed using Eqs. (8) and (9)^10^**.**

8 $${\upeta }_{\text{i}}=\frac{{\dot{\text{q}}}_{\text{u}}}{\left({\text{A}}_{\text{c}}\text{S}\right)}= \frac{\dot{\text{m}}{\text{ C}}_{\text{p}}}{{\text{A}}_{\text{c}}} \left[\frac{{\text{T}}_{0}-{\text{T}}_{\text{i}}}{\text{S}}\right]$$

9 $${\upeta }_{{\text{i}}} = {\text{F}}_{{\text{R}}} \left( {{\uptau \upalpha }} \right) - ({\text{F}}_{{\text{R}}} {\text{U}}_{{\text{l}}} )\left[ {\frac{{{\text{T}}_{{\text{i}}} - {\text{T}}_{{\text{a}}} }}{{\text{S}}}} \right]$$

### Uncertainty analysis

Standard uncertainty, obtained from calibration data and manufacturer specs, ensures reliable results by accounting for instrument limitations and calibration errors. It is expressed as a standard deviation or expanded uncertainty, typically with a coverage factor. Crucial for assessing the accuracy and precision of measurements, ensuring trustworthy results for further analysis. The standard uncertainty of the measuring device is given by following Eq. (10).

10 $$\text{Standard uncertainty }=\frac{\text{Precision measurement value}}{\sqrt{3}}$$

The uncertainty of the solar collector's instantaneous efficiency is given by Eq. (11)^17,26,27^

11 $${\Delta\upeta }_{\text{i}}=\sqrt{\left\{{\left[\frac{\partial\upeta }{\partial {\text{F}}_{\text{R}}} {\Delta \text{F}}_{\text{R}}\right]}^{2}+{\left[\frac{\partial\upeta }{\partial {\text{U}}_{\text{l}}} {\Delta \text{U}}_{\text{l}}\right]}^{2}+{\left[\frac{\partial\upeta }{\partial {\text{T}}_{\text{i}}} {\Delta \text{T}}_{\text{i}}\right]}^{2}+{\left[\frac{\partial\upeta }{\partial {\text{T}}_{\text{a}}} {\Delta \text{T}}_{\text{a}}\right]}^{2}+{\left[\frac{\partial\upeta }{\partial \text{s}} \Delta \text{s}\right]}^{2}\right\}}$$

The instantaneous solar efficiency was determined with an overall uncertainty of 4.5%. This uncertainty was computed by considering the individual uncertainties of the flow meter ± 4.1%, thermocouples ± 0.5 °C, anemometer ± 3% and pyranometer (± 5.5%).

## Result and discussion

### Water as the working fluid

**Figure 3:**
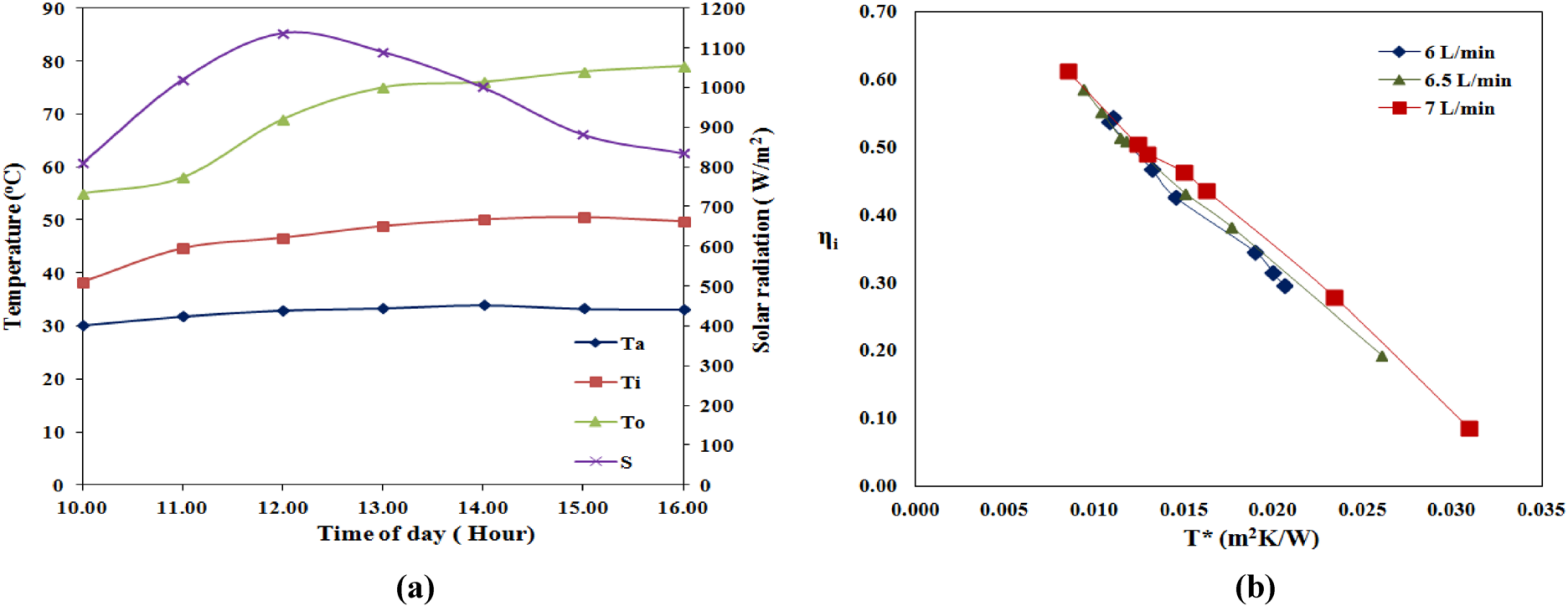
The measurement of temperatures, solar radiation and instantaneous efficiency with respect to time of the day (**a**) Experimental measurements of T_a_, T_i_, T_o_ & S concerning time of day at 6 L/min water flow rate as working fluid (**b**) Instantaneous solar collector efficiency concerning T* at a different volume flow rate.

The experiments were meticulously conducted from March to April 2022, specifically from 10:00 to 16:00, to gather data on the performance of a solar thermal system. Figure 3a provides a graphical representation of the temporal changes in solar radiation intensity and temperatures, including ambient, fluid inlet, and outlet temperatures, for distilled water as the base fluid at a constant volume flow rate of 6 L/min. The solar radiation intensity and temperatures were also diligently recorded for different volume flow rates, namely 6, 6.5, and 7 L/min, to comprehensively assess their impact on the system's behavior. The collected data were rigorously evaluated in accordance with the ASHRAE standard. The analysis revealed that the highest temperature variations in ambient, inlet, and exit temperatures were determined to be 0.7 °C, 0.5 °C, and 0.6 °C, respectively, over each test period. Moreover, the solar radiation increased in intensity until 12:00–13:00, followed by a decrease. These findings provide valuable insights into the system's performance under different operating conditions and can contribute to developing more efficient solar thermal systems.

Figure 3b represents the Instantaneous efficiency of solar collector efficiency with reduced temperature parameters, ${\text{T}}^{*}=\frac{\left({\text{T}}_{\text{i}}-{\text{T}}_{\text{a}}\right)}{\text{S}}$ at different volume flow rates. The readings were plotted as linear equations, and the values of F_R_U_l_ and F_R_(τα) were obtained by fitting the data into linear equations for different volume flow rates. The efficiency parameters, F_R_U_l_ and F_R_(${\uptau \upalpha }$), at each volume flow rate, were conveyed in Table 3. A lower value of F_R_U_l_ = 22.38 and a higher value of F_R_(τα) = 0.7959 is obtained at 7 L/min. This indicates a lower friction factor, which decreases pressure drop across the absorber tube and improves transmissivity in the absorber tube. This increases the instantaneous efficiency of solar collectors. A lower value of F_R_U_l_ = 22.38 and a higher value of F_R_(τα) = 0.7959 is obtained at 7 L/min. This indicates a lower friction factor, which decreases pressure drop across the absorber tube and improves transmissivity in the absorber tube. This, in turn, resulted in increased instantaneous efficiency of solar collectors. Furthermore, it was observed that higher volume flow rates positively influenced the solar collector efficiency.

**Table 3:** F_R_U_l_ and F_R_(τα) of solar parabolic collector for distinctive water flow rates.

Flow rate (L/min)	F_R_U_l_	F_R_(τα)	R^2^
6	24.23	0.7957	0.988
6.5	23.47	0.7954	0.995
7	22.38	0.7959	0.996

### Effect of surfactant on the stability of MWCNT nanofluids

The nanofluid was prepared without using a surfactant. In this case, the MWCNT nanoparticles were settled after a few minutes. The MWCNT nanoparticles were attributed to hydrophobic behavior and formed strong Vander Waals forces between them. The addition of surfactant to MWCNT will increase the stability. For the preparation of CNT nanofluid, Gum Arabic, Triton X-100, Sodium Deoxycholate, Humic Acid, Sodium Dodecyl Benzenesulfonate (SDBS), etc. were used as a surfactant^28^. Among these surfactants, SDBS gives the most promising results. Hence, nanofluids were prepared by adding an effective concentration of SDBS surfactant. This leads to modification on the surface of MWCNT and behaves in hydrophilic nature. The effects of the surfactant enhance the repulsive forces between the MWCNT particles to avoid the agglomerates and remain stable^29^. The stability of MWCNT was measured through the photographic method and was stable for more than a month. The long-term stability of MWCNTs was again characterized by centrifugation methods for 30 min at 3000 rpm. The results remained the same with the photographic method. This stable nanofluid was performed more effectively, resulting in solar parabolic collector efficiency.

### Effect of different concentrations of MWCNT nanoparticles in the water

**Figure 4:**
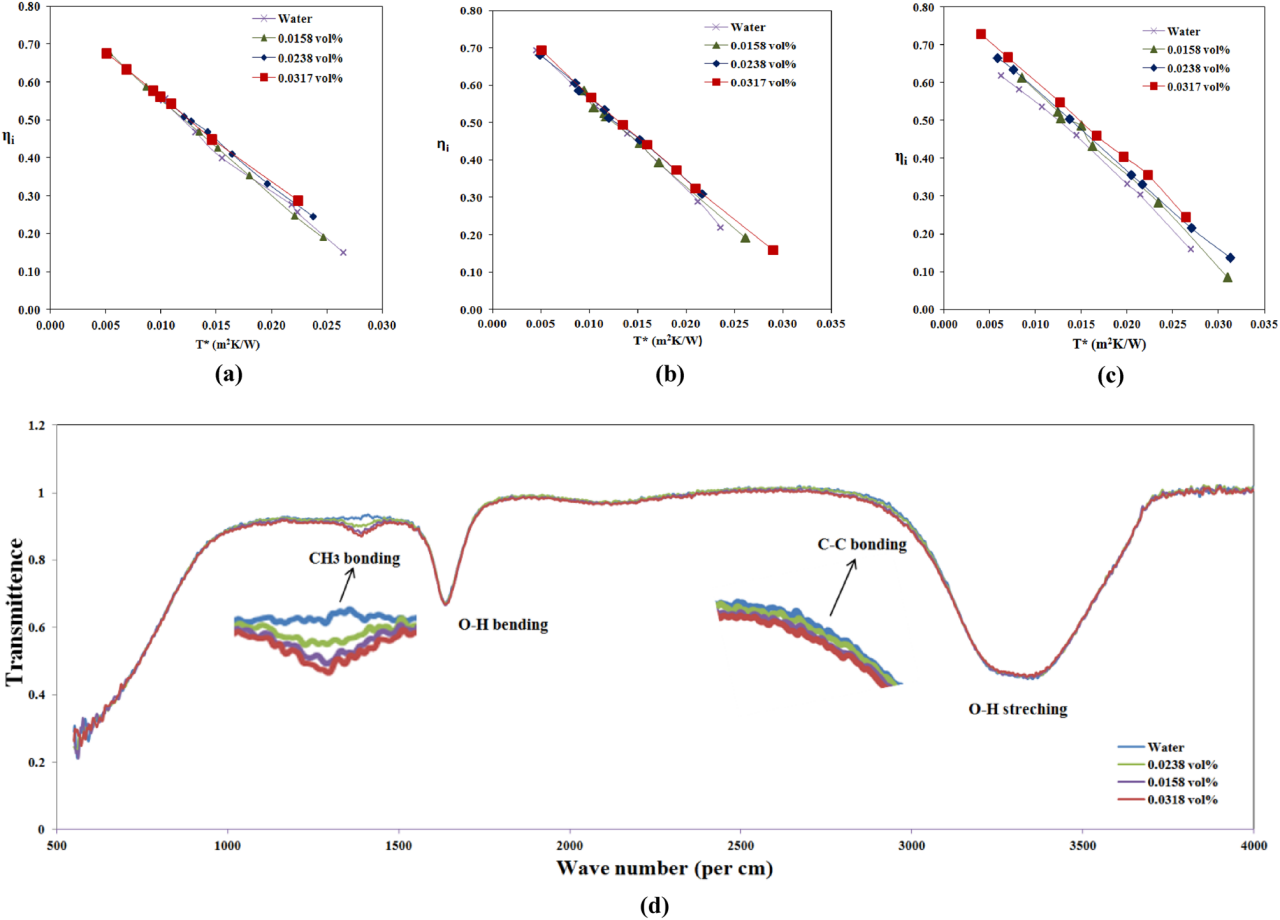
Effect of different concentrations of MWCNT nanoparticles in the water (**a**), (**b**) & (**c**) Instantaneous efficiency of collector η_i_ vs. T* at 6, 6.5 and 7 L/min, respectively and (**d**) FTIR spectroscopy of water and nanofluids.

The nanofluid is prepared with different percentages of volume concentrations such as 0.0158, 0.0238, and 0.0317% and SDBS surfactant is mixed with a base fluid to improve stability. The impact of variation of volume concentrations on instantaneous efficiency for different reduced temperatures $\frac{\left({\text{T}}_{\text{i}}-{\text{T}}_{\text{a}}\right)}{\text{S}}$ at flow rates of 6, 6.5 and 7 L/min is shown in Fig. 4a–c. The solar parabolic collector's instantaneous efficiency increased with volume fraction for all volume flow rates. For a flow rate of 6 L/min at a volume fraction of 0.0158%, collector instantaneous efficiency increases by 2% compared to the base fluid. The efficiency increases by 3% and 4% for 0.0238% and 0.0317% volume fractions, respectively. At a flow rate of 6.5 L/min, the instantaneous efficiency of the collector increased by 3% compared to distilled water, when a volume fraction of 0.0158%. Similarly, at the same flow rate, the efficiency improved by 4% and 7% for volume fractions 0.0238% and 0.0317%, respectively. At a flow rate of 7 L/min, 6% instantaneous efficiency magnified with 0.0158% volume fraction. The efficiency further positively dominated 8% and 11% at a volume fraction of 0.0238% and 0.0317%. The diffusion of nanoparticles in the base fluid significantly enhances the thermal conductivity of the nanofluid, resulting in improved heat transfer characteristics^30^. This increased thermal conductivity allows for more efficient heat dissipation, leading to enhanced performance of the solar parabolic collector.

Carbon nanotubes (CNTs) are renowned for their exceptional thermal conductivity, which surpasses that of other nanoparticles. These nanotubes, with diameters ranging from 5 to 15 nm, boast a unique cylindrical shape that results in a larger surface area for efficient heat transfer, making them highly desirable for various applications^24^. Moreover, when CNTs are used in lower volume fractions in a base fluid, they exhibit Newtonian fluid behavior, flowing smoothly without significant changes in viscosity. This characteristic is particularly advantageous in applications such as solar parabolic collectors, where optimal heat transfer is crucial for maximum performance. Furthermore, the Brownian motion mechanism, wherein CNTs exhibit random motion due to thermal fluctuations, can enhance the efficiency of solar parabolic collectors when employed in nanofluids.

However, the nanofluid becomes denser at higher volume fractions, leading to nonlinear behavior. As the volume fraction of CNTs increases, the attractive Vander Waals forces between nanoparticles become stronger than the Brownian motion, resulting in decreased stability and reduced performance of the solar parabolic collector. Therefore, in the present research, the volume concentration of CNTs is deliberately kept in a lower range to achieve the best results at a lower cost, ensuring optimal performance and stability in the solar parabolic collector.

In Fig. 4d, FTIR spectroscopy revealed important information about the atomic bonding in the nanofluid. The peak at near 1450 cm^−1^ indicated symmetric carbon-hydrogen bonding, which is present in the SDBS surfactant used in the nanofluid, and the absorption energy increased with surfactant concentration. Peaks at nearly 1600 and 3300 cm^−1^ indicated oxygen and hydrogen bonding from water molecules in bending and stretching modes, respectively. The weak carbon–carbon bonding peaks near 2800–2900 cm^−1^ indicated the presence of carbon atoms from MWCNTs, with higher concentrations leading to enhanced energy absorption by the working fluid^31^.

However, it's important to note that energy absorption may vary due to environmental factors, such as solar intensity, absorber surface temperatures, and working fluid inlet temperatures. A decreased temperature difference between the absorber tube surface and the working fluid may reduce heat gain. Based on research findings, nanofluids have the potential to achieve energy gains ranging from 1000 to 1220 Joules per hour for a 20-L storage capacity. These values represent the potential heat energy that can be gained from the system when MWCNT nanoparticles are dispersed in the base fluid.

### The Influence of volume flow rates on solar parabolic collector efficiency

**Figure 5:**
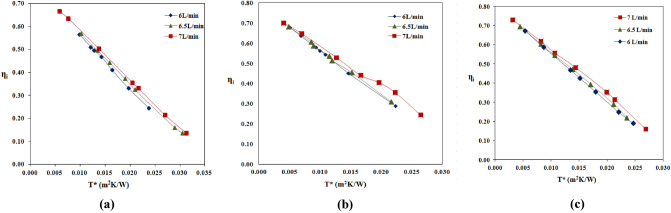
The influence of volume flow rates on solar parabolic collector efficiency (**a**), (**b**) & (**c**). Instantaneous efficiency of collector η_i_ vs. T* at distinct volume flow rates for 0.0158, 0.0238 & 0.0317 vol% nanofluids.

The volume flow rates of nanofluids varied as 6, 6.5 and 7 L/min by a regulating valve. For every volume flow rate, the instantaneous efficiency of the collector vs. the reduced temperature parameters ${\text{T}}^{*}=\frac{\left({\text{T}}_{\text{i}}-{\text{T}}_{\text{a}}\right)}{\text{S}}$ changes are established. Figure 5a–c shows the variations of 0.0158, 0.0238 and 0.0317 vol% nanofluids at 6, 6.5 and 7 L/min, respectively. In all the volume concentrations of nanofluids, the solar collector efficiency is magnified with incremental volume flow rates. As the flow rate of working fluid increases temperature gradient decreases, and this develops a high heat transfer coefficient. This is a reduction in temperature difference between T_i_ and T_a_ (temperature gradient) and forms a smaller value of the T* parameter, so solar collector efficiency increases^3,8,32^. A previous study by researcher Lyudmila Knysh on numerical method was conducted with MWCNT-water nanofluid and achieved nearly 10% improvement in the solar collector efficiency^33^.

## Conclusions

The experimental work is conducted on solar parabolic collectors per ASHRE standards for various volume fraction% nanofluids with different flow rates. From the experimental result, collector efficiency increases with increasing volume flow rates. At a high flow rate, the temperate difference between the absorber tube and the working fluid is minimum; hence more heat is transferred. Adding a sufficient quantity of SDBS surfactant to nanofluid will improve the Brownian motion of MWCNT nanoparticles. This results in the effective stability of the nanoparticles and improves heat transfer. As incremental volume concentration of MWCNT in a base fluid increases collector efficiency but at the same viscosity of the working fluid increases, affixing nanoparticles to the base fluid has certain limitations. In the present study, at 7 L/min with 0.0317 vol%, nanofluids have the highest collector efficiency. The solar collector efficiency was marginally enhanced by 10 to 11% in the present experimental work.

## Data Availability

The datasets used and analyzed during the current study are available from the corresponding author upon reasonable request.

## List of symbols

A: Ambient
${\text{A}}_{\text{c}}$: Area of the collector (m^2^)
bf: Base fluid
C: Concentration ratio
CNT: Carbon nanotube
C_p_: Specific heat (J/kg K)
D: Diameter (m)
FTIR: Fourier Transform infrared
i: Fluid inlet (°C)
o: Fluid outlet (°C)
F_R_: Collector heat removal factor
k: Thermal conductivity (W/mK)
L: Length of the collector (m)
L/h: Liter per hour
L/min: Liter per minutes
m: Mass (kg)
$\dot{\text{m}}$: Mass flow rate (kg/s)
min: Minutes
MWCNT: Multi-walled carbon nanotube
nf: Nanofluid
Np: Nanoparticle
${\dot{\text{q}}}_{\text{u}}$: Useful energy gain (W)
rpm: Revolution per minute
S: Global solar radiation (W/m^2^)
SDBS: Sodium Dodecyl-Benzene-sulfonate
T: Temperature (°C)
T*: Reduced temperature (K-m^2^/W)
TEM: Transmission Electron Microscopes
U_l_: Overall coefficient loss (W/m^2^K)
W: Width of the collector (m)
ρ: Density (kg/m^3^)
Φ: Volume concentration (vol%)
µ: Dynamic viscosity (N-s/m^2^)
$${\upeta }_{\text{i}}$$: Instantaneous efficiency
${\uptau \upalpha }$: Transmission-absorption factor
